# Switching protease inhibitors to rilpivirine in HIV‐positive individuals with complete viral suppression and without prior HIV drug resistance in a resource‐limited setting: a randomized controlled trial

**DOI:** 10.1002/jia2.25462

**Published:** 2020-04-11

**Authors:** Kulissara Palanuphap, Somnuek Sungkanuparph

**Affiliations:** ^1^ Department of Medicine Faculty of Medicine Ramathibodi Hospital Mahidol University Bangkok Thailand; ^2^ Chakri Naruebodindra Medical Institute Faculty of Medicine Ramathibodi Hospital Mahidol University Samut Prakan Thailand

**Keywords:** rilpivirine, protease inhibitor, switching, antiretroviral therapy, randomized controlled trial

## Abstract

**Introduction:**

Prior to the availability of rilpivirine (RPV), patients who could not tolerate efavirenz and nevirapine (NVP) were treated with protease inhibitor (PI)‐based antiretroviral therapy (ART). Dyslipidaemia and other metabolic complications are commonly associated with PI use. This study aimed to compare the efficacy and adverse events between switching from PI‐based to RPV‐based regimen, versus continuing PI‐based regimens in HIV‐positive individuals with complete viral suppression.

**Methods:**

A randomized controlled trial was conducted in HIV‐positive individuals receiving PI‐based regimens with undetectable HIV RNA and without prior HIV drug resistance. Patients were enrolled between July and December 2017 in a university medical centre in Bangkok, Thailand. They were randomized to switch from PIs to RPV (switch group) or continue ritonavir‐boosted PI (control group). Primary endpoint was the proportion of patients with undetectable HIV RNA at 48 weeks. Changes in CD4 cell counts, lipid profiles and adverse events were also analysed.

**Results and discussion:**

A total of 84 patients were enrolled, 42 in each group. Mean age was 47.7 years and 53.6% were males. At 48 weeks, 95.2% of patients in the switch group and 92.9% of control group had maintained undetectable HIV RNA (difference rate 2.4%; 95% CI, −9.6 to 14.7). Means of CD4 cell counts were 611 and 641 cells/mm^3^ in switch and control groups respectively (*p* = 0.632). Mean changes in lipid profiles (switch vs. control groups) were: total cholesterol, −12.5 versus + 12.2 (*p* = 0.024); LDL, −3.4 versus + 6.2 (*p* = 0.040); HDL, +1.6 versus + 1.9 (*p* = 0.887); and triglycerides, −82.6 versus − 24.4 mg/dL (*p* = 0.031). The mean changes of glucose and eGFR were similar (*p *> 0.05) between the two groups. The mean change of ALT was significantly greater in switch group (18.2 vs. 4.0 U/L, *p* = 0.017). One patient in switch group had anorexia and elevated ALT at 14 weeks and completely recovered after RPV discontinuation.

**Conclusions:**

Switching PIs to RPV, in patients with complete viral suppression and without prior HIV drug resistance, sustains viral suppression and yields better lipid profiles. This finding supports its use as switching therapy in patients receiving PI‐based regimens due to intolerance to efavirenz and NVP and previous alternatives limited to PI in resource‐limited settings.

## 1 Introduction

Scaling‐up of antiretroviral therapy (ART) has resulted in significant reductions in morbidity and mortality in people living with HIV (PLHIV) worldwide, including those in resource‐limited settings [1, 2, 3, 4]. The World Health Organization and national AIDS programmes in many developing countries have recommended tenofovir/emtricitabine/efavirenz (TDF/FTC/EFV) as a preferred first‐line regimen [5] due to its efficacy and tolerability. Nevirapine (NVP) has been the alternative to EFV in resource‐limited settings [5, 6, 7]. However, some patients experience adverse drug effects from both EFV and NVP. Prior to the availability of other non‐nucleoside reverse‐transcriptase inhibitors (NNRTIs) and integrase inhibitors, alternatives were limited to ritonavir‐boosted protease inhibitor (PI)‐based ART in resource‐limited countries. HIV‐positive individuals who could not tolerate both EFV and NVP were treated with PI‐based regimens [5]. A significant number of PI‐associated lipid abnormalities have been reported [8, 9, 10]. These alterations in lipid values, including elevation of total cholesterol (TC) and low‐density lipoprotein cholesterol (LDL) levels, are known to be major risk factors for cardiovascular disease [11].

Rilpivirine (RPV) is a once‐daily NNRTI, given at a daily dose of 25 mg which can be co‐formulated with two nucleoside reverse‐transcriptase inhibitors (NRTIs) [12]. The regimen of tenofovir/emtricitabine (TDF/FTC)/RPV is categorized as a recommended initial regimen for HIV‐positive individuals with pretreatment HIV RNA <100,000 copies/mL and a CD4 count >200 cells/mm^3^ in both the United States and the European guidelines [12, 13]. RPV has shown non‐inferior efficacy compared with EFV in treatment‐naïve HIV‐positive individuals with HIV RNA <500,000 copies/mL, along with a favourable safety and tolerability profile [14, 15, 16]. In addition, an RPV‐based regimen is also more convenient for patients and more affordable in resource‐limited countries when compared to PIs. Currently, RPV is available in resource‐limited settings and could be considered as an alternative for switching therapy in HIV‐positive individuals who have complete viral suppression with PI‐based regimens and have no prior history of HIV drug resistance to NNRTIs. Therefore, this study aimed to compare the efficacy and adverse events between switching from a PI‐based regimen to an RPV‐based regimen versus continuing a PI‐based regimen in HIV‐positive individuals with complete viral suppression and without prior HIV drug resistance.

## 2 Methods

A randomized controlled trial was conducted in HIV‐positive individuals receiving a ritonavir‐boosted PI‐based ART with undetectable HIV RNA (<40 copies/mL), and without prior HIV drug resistance. Patients were consecutively screened and enrolled between July and December of 2017 in a university medical centre in Bangkok, Thailand, and followed up for 48 weeks. All the study patients had documented HIV infection and met the following criteria: (1) >18 years of age, (2) receiving a PI‐based ART (due to intolerance to EFV and NVP, and alternatives were limited to PI‐based regimens) for at least 12 months, (3)  aving undetectable HIV RNA (<40 copies/mL) at screening, and (4) able to sign an informed consent form. Patients were excluded if they had a history of virologic failure and/or HIV drug resistance, used other drugs that might interact with RPV, and female patients during pregnancy or breastfeeding. To determine that there was no prior HIV drug resistance, each patient needed to have no history of virologic failure during treatment and had no primary HIV drug resistance prior to starting ART. All patients in this study had been performed an HIV genotypic resistance test as previously published [17]. Written informed consent was obtained from all eligible patients before randomization. The study was approved by the institutional review board, the Committee on Human Rights Related to Research Involving Human Subjects, Faculty of Medicine Ramathibodi Hospital, Mahidol University.

All the enrolled patients were randomly assigned (1:1) by computer‐generated random numbers, to switch from a ritonavir‐boosted PI to RPV (switch group), or to continue the current PI‐based regimen (control group). RPV was taken with a regular meal. All patients were prospectively followed up for 48 weeks. The laboratory assessments were performed at baseline, weeks 24 and 48. Laboratory tests included a complete blood count, CD4 cell count, chemistry panel [e.g. alanine aminotransferase (ALT), creatinine, fasting sugar and lipid panel] and urinalysis. HIV RNA was performed using Amplicor HIV‐1 Monitor Test version 1.5 (Roche, Basel, Switzerland).

The primary outcome was the proportion of patients with undetectable HIV RNA (<40 copies/mL) at 48 weeks. The secondary outcomes were the mean changes of CD4 cell count and lipid levels including TC, LDL, high‐density lipoprotein cholesterol (HDL) and triglycerides (TG) and adverse events between the two groups during the study. Virologic failure was determined when HIV RNA was ≥200 copies/mL based on two consecutive measurements. We classified patients with missing HIV RNA measurements as having virologic failure at each time point for which a value was not available (“missing = failure”). Adverse events were defined as any undesirable experience associated with the use of antiretroviral drugs included rash, gastrointestinal symptoms (nausea, vomiting and epigastric pain), neurological symptoms and psychiatric events. Serious adverse events included any untoward medical occurrence that resulted in death, life‐threatening conditions, hospitalization or prolongation of existing hospitalization, or persistent or significant disability.

From our infectious disease clinic of approximately a two thousand HIV‐positive population, 75% of persons receiving PI‐based regimens have complete viral suppression at 48 weeks of ART. Sample size was calculated from the proportional response rates from a previous trial [14, 15]. A population of 84 (42 in each group) was required to establish non‐inferiority of the switch group compared to the control group, at 0.8 power, and a 0.05 significance level. The analysis was based on intention‐to‐treat (ITT) populations. For the primary efficacy endpoint, non‐inferiority of switching PIs to RPV could be claimed if the lower 95% confidence limit for the difference in efficacy was not below the prespecified non‐inferiority margin (non‐inferiority limit) of −12%. Secondary outcomes were compared using *t* test or Mann‐Whitney *U* test for continuous variables and chi square or Fisher’s exact tests for categorical variables. All analyses were performed using an electronic database organized in SPSS version 18.0. A *p* < 0.05 was considered statistically significant.

## 3 Results and Discussion

**Figure 1:**
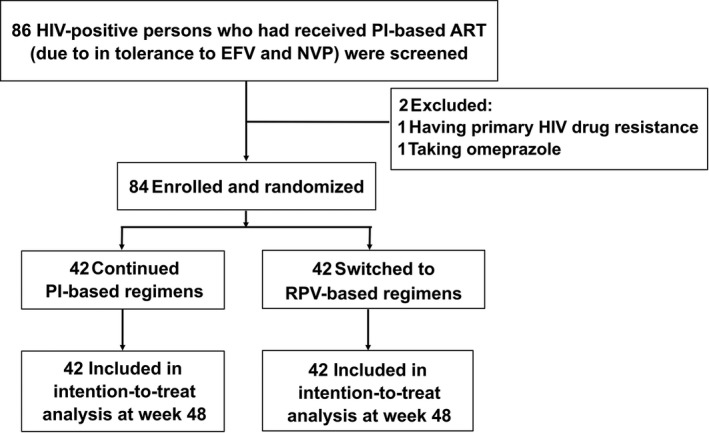
Flowchart of study patient enrolment.

Of the 86 patients screened, 84 fulfilled inclusion and exclusion criteria and were enrolled, 42 in each group (Figure 1). The mean age was 47.7 years and 53.6% of patients were males. The mean baseline CD4 cell count was 609 cells/mm^3^. Baseline characteristics including age, gender, body weight, duration of ART, distribution of NRTIs and PIs used, CD4 cell count, lipid profiles, glucose, ALT and estimated glomerular filtration rate (eGFR) between the two groups were similar, as summarized in Table 1.

**Table 1:** Baseline characteristics of patients in switch and control groups

Characteristics	Switch group	Control group
Age, years, mean ± SD	48.1 ± 10.3	47.2 ± 8.1
Sex, number (%)		
Male	21 (50.0)	24 (57.1)
Female	21 (50.0)	18 (42.9)
Body weight, kg, mean ± SD	62.6 ± 12.7	61.4 ± 12.8
Duration of ART, years, median (IQR)	8.8 (4.9 to 12.2)	9.2 (5.3 to 13.0)
PI‐based regimens, number (%)		
Atazanavir/ritonavir	26 (61.9)	28 (66.7)
Lopinavir/ritonavir	15 (35.7)	12 (28.6)
Darunavir/ritonavir	1 (2.4)	2 (4.7)
NRTI backbone, number (%)		
Tenofovir + Lamivudine or Emtricitabine	31 (73.8)	28 (66.7)
Abacavir + Lamivudine	4 (9.5)	5 (11.9)
Zidovudine + Lamivudine	7 (16.7)	6 (14.3)
Others	–	3 (7.1)
CD4 cell count, cells/mm^3^, mean ± SD	616 ± 235	601 ± 244
Lipid profiles, mg/dL, mean ± SD		
Total cholesterol	198 ± 37	199 ± 32
HDL cholesterol^3^	45 ± 13	43 ± 12
LDL cholesterol	115 ± 28	112 ± 33
Triglycerides	185 ± 108	208 ± 148
ALT, U/L, mean ± SD	34.0 ± 20.8	30.4 ± 12.6
eGFR, mL/min, mean ± SD	91.1 ± 19.6	90.0 ± 17.1

ALT, alanine transaminase; ART, antiretroviral therapy; eGFR, estimated glomerular filtration rate; HDL, high‐density lipoprotein; IQR, interquartile range; LDL, low‐density lipoprotein; NNRTI, non‐nucleoside reverse transcriptase inhibitor; PI, protease inhibitor; SD, standard deviation.

**Figure 2:**
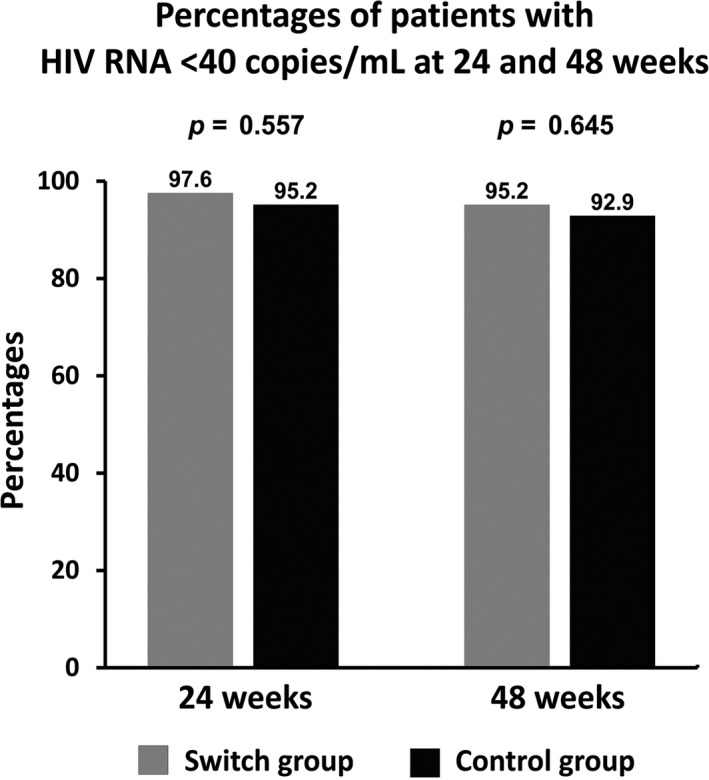
Intention‐to‐treat analysis for patients with viral suppression between switch and continue groups at 24 and 48 weeks.

**Figure 3:**
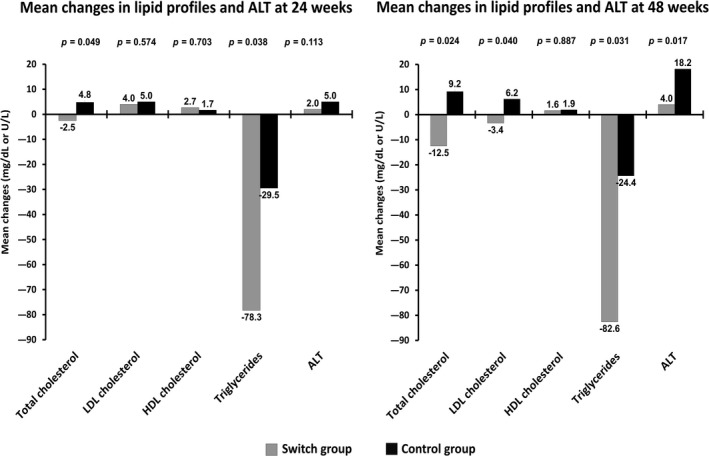
Mean changes in lipid profiles and ALT between switch and control groups at 24 and 48 weeks.

At 48 weeks, 95.2% of patients in the switch group and 92.9% of the control group had maintained undetectable HIV RNA (difference rate 2.4%; 95% CI, −9.6 to 14.7). This met the prespecified noninferiority criterion. The proportions of patients who maintained viral suppression at 24 and 48 weeks are shown in Figure 2. The means of CD4 cell counts were 611 and 641 cells/mm^3^ in the switch and control groups respectively (*p* = 0.632). The mean changes in lipid profiles (switch vs. control groups) were: TC, −12.5 versus +12.2 (*p* = 0.024); LDL, −3.4 versus +6.2 (*p* = 0.040); HDL, +1.6 versus +1.9 (*p* = 0.887); and TG, −82.6 versus −24.4 mg/dL (*p* = 0.031) (Figure 3). The mean changes of glucose and eGFR were similar between the two groups and the values were not significantly changed from baseline (*p *> 0.05). The mean change of ALT was significantly greater in the switch group compared to the control group (18.2 vs. 4.0 U/L, *p* = 0.017). A female patient in the switch group had anorexia and an elevated ALT of 65 U/L at 14 weeks after switching and completely recovered to be within normal range within two weeks after RPV discontinuation. The total and direct bilirubin were within normal ranges. An ultrasonography of the upper abdomen was performed and the result was unremarkable. The investigations for hepatitis A virus, hepatitis B virus, hepatitis C virus and hepatitis E virus were all negative. She had no history of alcohol or herbal medicine consumption.

PLHIV may have benefits from switching from a PI‐based regimen to an RPV‐based regimen, even when they have complete viral suppression. These include managing or preventing short‐term or long‐term adverse effects, high pill burden, problematic drug interactions or costs. [12, 18]. Although dolutegravir is currently recommended in many treatment guidelines [12, 13], it still not accessible in many resource‐limited countries. In contrast, RPV is widely available and a less expensive cost in resource‐limited settings. Switching PIs to RPV may benefit HIV‐positive individuals by reducing pill burden and decreasing the risk for long‐term PI‐related adverse effects such as dyslipidaemia and insulin resistance.

The present study has demonstrated that in HIV‐positive individuals taking a PI‐based ART with complete viral suppression and without prior HIV drug resistance, switching to an RPV regimen was not inferior to continuing a PI‐based ART, in term of maintained viral suppression at 48 weeks. The rates of sustained viral suppression in both treatment groups were also quite high. One of the key success factors was the fact that we enrolled only patients without a history of HIV drug resistance. This is important because the majority of patients taking a PI‐based ART in resource‐limited settings have failed their first‐line NNRTI‐based regimen. Those patients were not eligible for this study because RPV has significant cross‐resistance to EFV and NVP [19, 20]. The principal goal of ART regimen switching is to improve a patient’s quality of life while maintaining virologic suppression and not jeopardizing future options.

Immunological response in patients switching PIs to RPV was also similar between the two treatment groups. In this study, all patients at entry had complete viral suppression with a median duration of their first‐line ART of nine years and high CD4 cell counts. This reflects the good adherence on ART among these study patients. It has been established that good adherence on ART is associated with long‐term viral suppression and immune recovery [21, 22]. Although the rates of viral suppression were relatively high in both groups of this study, these rates slightly declined during the follow‐up period. Many reasons could contribute to this finding, such as drug‐drug and drug‐food interaction, and declining adherence to the medications [14, 15, 16].

In addition to sustained viral suppression and immune recovery, our study demonstrated that switching from PIs to RPV was associated with statistically significant improvement of TC, LDL and TG at 48 weeks. Improvement of TC and TG occurred significantly starting at the 24 week follow‐up. In a large Asian cohort, the development of fatal and nonfatal cardiovascular events was associated with high TC and TG [23]. This switching strategy may minimize some risks of cardiovascular diseases, one of leading causes of death in PLHIV in the ART era. Regarding adverse events of liver function, the mean change of ALT was significantly greater in the switch group compared to the control group and one patient in the switch group had clinical hepatitis. In various clinical trials, RPV showed a low rate of liver toxicity, similar to EFV in development studies [24]. Nevertheless, close monitoring of liver function after switching to RPV is recommended.

To the best of our knowledge, this is the first randomized controlled trial regarding switching ART from first‐line PI‐based regimens to an RPV‐based regimen in patients with complete viral suppression and without history of HIV drug resistance. The strength of our study was the study design of a randomized control trial that RPV was the only antiretroviral agent in the regimen that was switched. Therefore, the efficacy of RPV as a switch therapy has been demonstrated. However, there were some limitations. The study was not blinded to investigators and participants. Therefore, the open‐label design may actually be more reflective of true adherence to each particular regimen. This is instrumental to efficacy because PI‐based and RPV‐based regimens have great differences in pill burden, drug and food interactions, as well as directions for taking. These differences directly impact the ease of adherence and in turn may affect efficacy. In addition, a long‐term follow‐up study may be needed to demonstrate whether the benefit of lipid profile changes will translate into benefits for cardiovascular disease.

## 4 Conclusions

Switching PIs to RPV, in patients with complete viral suppression and without prior HIV drug resistance, sustains viral suppression and yields better lipid profiles. This finding supports its use as switching therapy in patients receiving PI‐based regimens due to intolerance to efavirenz and NVP and previous alternatives limited to PIs in resource‐limited settings. Although uncommon, elevation of liver enzymes leading to RPV discontinuation may occur. Close monitoring of liver function after switching is recommended.

## Competing interest

All authors declare no competing interests related to this work.

## Authors’ contributions

KP and SS performed the research and oversaw the participants. SS designed the research study. SS performed all statistical analysis. KP and SS wrote the paper. All authors critically reviewed and approved the final draft of manuscript.
